# DNA fragmentation index (DFI) as a measure of sperm quality and fertility in mice

**DOI:** 10.1038/s41598-020-60876-9

**Published:** 2020-03-02

**Authors:** Ming-Wen Li, K. C. Kent Lloyd

**Affiliations:** 1 Mouse Biology Program, University of California, Davis, CA 95618 USA; 2 Department of Surgery, School of Medicine, University of California (Davis), Sacramento, CA 95817 USA

**Keywords:** Embryology, Genetics research

## Abstract

Although thousands of genetically modified mouse strains have been cryopreserved by sperm freezing, the likelihood of cryorecovery success cannot be accurately predicted using conventional sperm parameters. The objective of the present study was to assess the extent to which measurement of a sperm DNA fragmentation index (DFI) can predict sperm quality and fertility after cryopreservation. Using a modified TUNEL assay, we measured and correlated the DFI of frozen-thawed sperm from 83 unique mutant mouse strains with sperm count, motility and morphology. We observed a linear inverse correlation between sperm DFI and sperm morphology and motility. Further, sperm DFI was significantly higher from males with low sperm counts compared to males with normal sperm counts (P < 0.0001). Additionally, we found that viable embryos derived using sperm from males with high DFI (62.7 ± 7.2% for IVF and 73.3 ± 8.1% for ICSI) failed to litter after embryo transfer compared to embryos from males with low DFI (20.4 ± 7.9% for IVF and 28.1 ± 10.7 for ICSI). This study reveals that measurement of DFI provides a simple, informative and reliable measure of sperm quality and can accurately predict male mouse fertility.

## Introduction

It is well known that normal embryonic development is dependent on the delivery of intact and complete genetic material from sperm to oocyte^1,2^. However, fragmentation of sperm nuclear DNA caused by multiple factors including errors in spermiogenesis, poor chromatin compaction, sperm apoptosis, endogenous caspases and endonucleases, oxidative stress, chemotherapeutic agents, radiation, infection, lifestyle, and other causes is commonly observed in sperm from humans and animals^1–3^. Numerous studies have attempted to assess the association between outcomes of assisted reproductive technologies (ART) and sperm DNA fragmentation (SDF) which has a negative effect on sperm quality and fertility^4–8^. However, due to variation between SDF assays and protocols, differences in study populations, lack of test standardization, systematic reviews and meta-analyses have been unable to make robust conclusions regarding the full impact of DNA fragmentation on sperm quality and fertility^9–19^. Therefore, today male fertility status is based on an assessment of conventional sperm parameters (count, motility and morphology) despite the inability to differentiate between infertile and fertile males^9,20,21^. Further studies are needed to clarify the exact role of sperm DNA damage within the myriad of other male and female factors contributing to reproductive outcomes after IVF (*in vitro* fertilization) and ICSI (intracytoplasmic sperm injection)^18,19^.

Because of their similarities with human anatomy, physiology and genetics, thousands of genetically modified mice have been used to study human physiology and diseases^22^. As a result, management of these burgeoning mouse numbers is increasingly reliant upon sperm cryopreservation and recovery by ART such as IVF and ICSI followed by embryo transfer^23^. Examinations used today to assess sperm quality in mice before and after cryopreservation include an evaluation of sperm count, motility and morphology^24^. However, as in humans, conventional sperm parameters cannot reliably be used to predict male reproductive success following ART. In our experience, non-genetic causes of infertility and subfertility, repeated IVF failures, non-surgical causes of embryo transfer failure, early gestational death, and other indeterminate causes of pregnancy loss observed in genetically modified mouse strains occur not infrequently despite normal sperm count, motility and morphology. Therefore, more reliable means to accurately assess sperm quality and predict male fertility are needed in order to aid the selection of male mice for cryopreservation and the most appropriate assisted reproductive techniques for cryorecovery.

Sperm DNA integrity is essential for embryo development^25–27^, although sperm with minimal DNA damage retain fertilizing ability^28^. Limited studies in animals and humans have shown that sperm DNA can be damaged by cryopreservation^26,29–32^, and there is a growing concern about the impact of cryodamage on ART outcome. Thus, the objectives of the present study were to study (1) the effects of cryopreservation on sperm DNA integrity, (2) the correlation between post-thaw sperm DNA fragmentation index (DFI) as a measure of sperm DNA fragmentation and conventional sperm parameters (count, motility and morphology), and (3) the relationship between post-thaw sperm DFI and fertility in mice. To avoid female factor infertility, wildtype oocytes from the same wildtype mouse strain and vendor were used for all IVF and ICSI procedures.

## Results

### Effects of cryopreservation on sperm motility and DNA integrity

**Figure 1:**
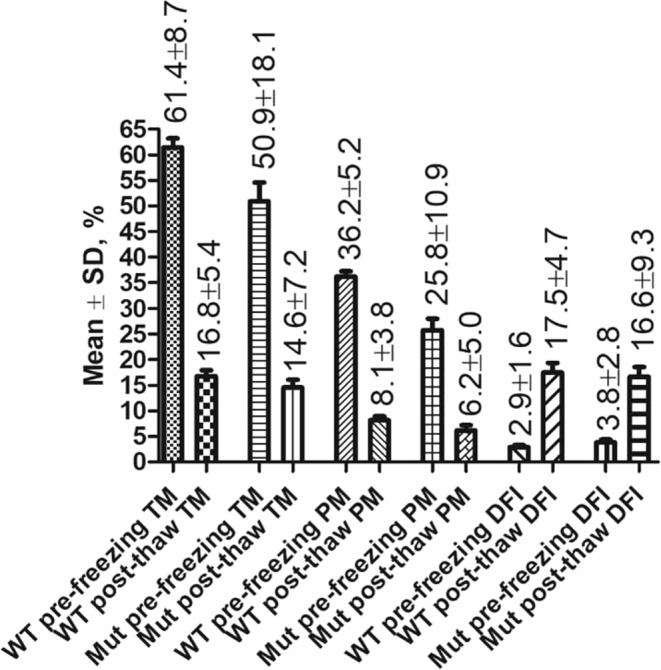
Cryopreservation significantly (P < 0.001) reduced sperm total motility (TM) and progressive motility (PM) and increased sperm DFI in both wildtype (WT) C57BL/6N males (n = 24) and mutant (Mut) males (n = 24 representing 24 mouse strains). P < 0.0001 between pre-freezing and post-thaw in TM, PM and DFI in both WT and Mut strains.

To determine the extent to which sperm motility and DNA integrity are affected by cryopreservation, total motility, progressive motility and DFI of sperm from 24 wildtype C57BL/6N males and 24 mutant males proven fertile by natural mating (1 male per mutant mouse strain) were assessed before and after cryopreservation. The data in Fig. 1 show that cryopreservation reduced sperm total motility and progressive motility (P < 0.0001) and increased sperm nuclear DNA fragmentation level (P < 0.0001) significantly compared with controls in both wildtype and mutant mouse strains.

### Correlation between sperm DFI and motility in mutant males

**Figure 2:**
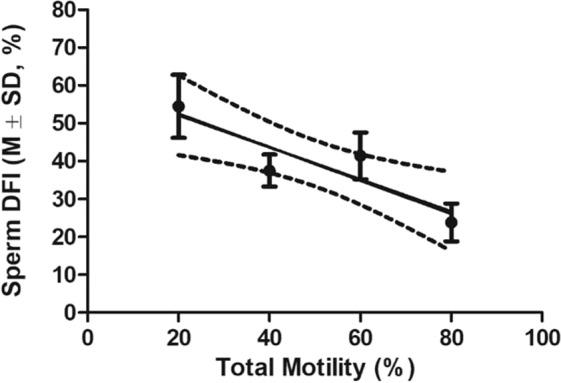
The linear correlation between DFI of frozen-thawed sperm and sperm total motility. Pearson r = −0.9014, P < 0.05. Total n = 60. Total motilities were sorted into 6–20%, 21–40%, 41–60% and 61–80% for measuring the correlation.

**Figure 3:**
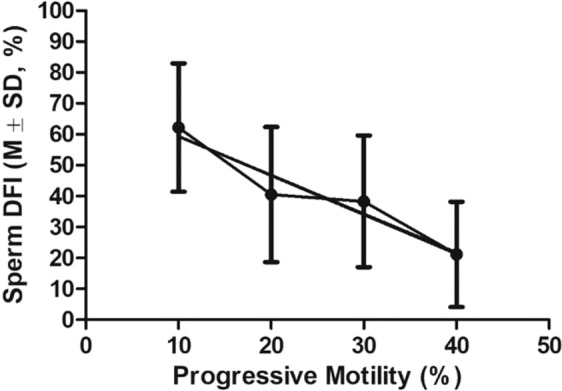
The linear correlation between DFI of frozen-thawed sperm and sperm progressive motility. Pearson r = −0.9615, P < 0.05. Total n = 60. Progressive motilities were sorted into 1–10%, 11–20%, 21–30% and 31–48% for measuring the correlation.

As shown in Fig. 1, total (14.6 + 7.2%) and progressive (6.2 ± 5.0%) motility of cryopreserved sperm from fertile wildtype and mutant males, respectively, were low and largely variable. Therefore, to determine the relationship between post-thaw sperm DFI and motility we performed a correlation analysis using pre-freezing sperm motilities of mutant males for 60 mutant strains. We found that sperm DFI was inversely correlated with both total motility (r = −0.90, Fig. 2) and progressive motility (r = −0.96, Fig. 3).

**Figure 4:**
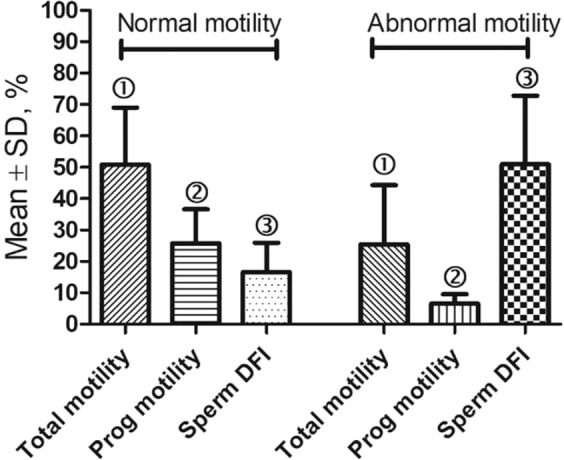
Comparison of fresh sperm motilities and DFI of frozen-thawed sperm in a group of mutant males with normal fertility (n = 24) and that in a group of mutant males with impaired motility (n = 17). P < 0.0001 between bars with the same symbol ➀ or ➂, and P < 0.001 between bars with the same symbol ➁.

We then compared the DFI of frozen-thawed sperm from the 24 fertile mutant males mentioned above with that of 17 mutant males with impaired motility (Fig. 4). As expected, the frozen-thawed sperm DFI (51.0 ± 21.8%) was significantly higher (P < 0.0001) in males with poorer motility (total motility 25.5 ± 18.8% and progressive motility 6.53 ± 3.0%) compared to the frozen-thawed sperm DFI (16.6 ± 9.3%) of males with normal motility (total motility 50.9 ± 18.1% and progressive motility 25.8 ± 10.9%, respectively).

### Correlations between post-thaw sperm DFI and sperm morphology in mutant males

**Figure 5:**
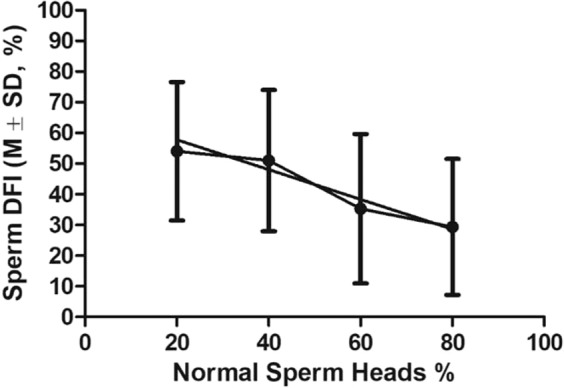
The linear correlation between DFI of frozen-thawed sperm and % of sperm with normal head morphology. Pearson r = −0.9678, P < 0.05. Total n = 60. Sperm head percentages were sorted into 0–29%, 30–49%, 50–70%, and 71–95% for measuring the correlation.

**Figure 6:**
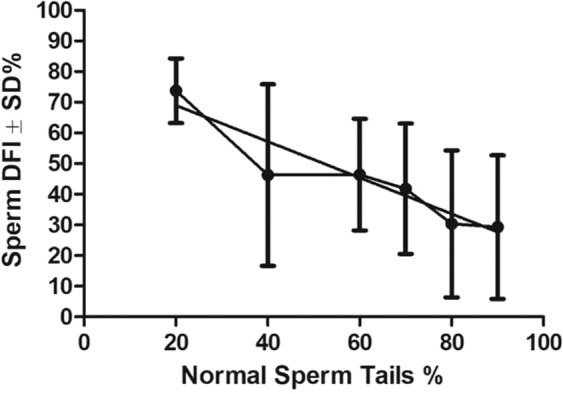
The linear correlation between DFI of frozen-thawed sperm and % of sperm with normal tail morphology. Pearson r = −0.9376, P < 0.05. Total n = 60. Normal sperm tail percentages were divided into 10–20%, 30–50%, 51–60%, 61–70% and 81–95 for measuring the correlation.

To determine the correlation between DFI and sperm morphology (head and tail), we analyzed frozen-thawed sperm from the 60 males representing 60 mutant mouse strains mentioned above. There were strong statistically significant (p < 0.05) inverse correlations between sperm DFI and normal sperm head (r = −0.9678, Fig. 5) and tail (r = −0.9376, Fig. 6) morphologies.

**Figure 7:**
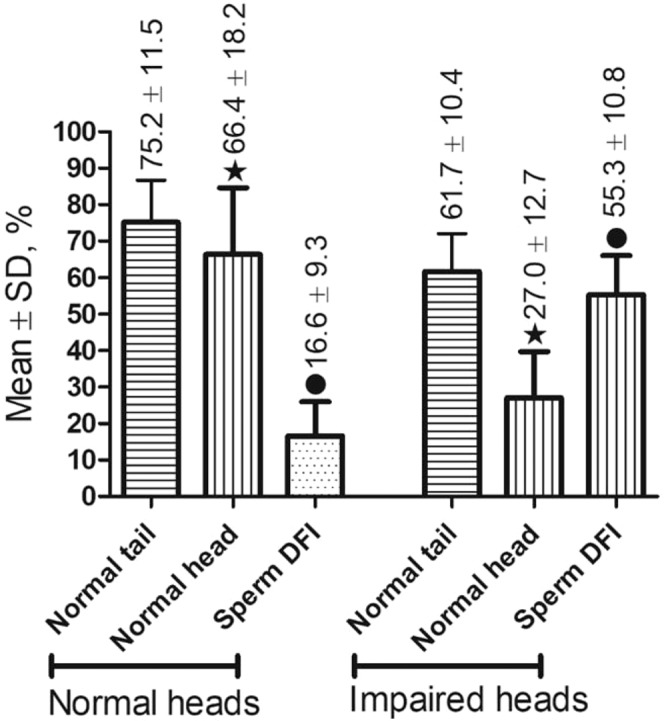
Comparison of sperm morphology and DFI of frozen-thawed sperm in a group of mutant males with normal fertility (the control, n = 24) and that in a group of mutant males with impaired sperm head morphology (n = 17). P < 0.0001 between bars with the same symbol.

We then compared the DFI of frozen-thawed sperm from males from 24 fertile mutant strains with that of males representing 9 mutant mouse strains with a significantly greater (p < 0.0001) proportion of abnormal sperm heads (Fig. 7). As expected, we found that the average sperm DFI (55.3 ± 10.6%) of males with a high proportion of abnormal sperm heads was significantly greater (p < 0.0001) than the sperm DFI (16.6 ± 9.3%) of normal males.

### Correlation between post-thaw sperm DFI and sperm count in mutant males

**Figure 8:**
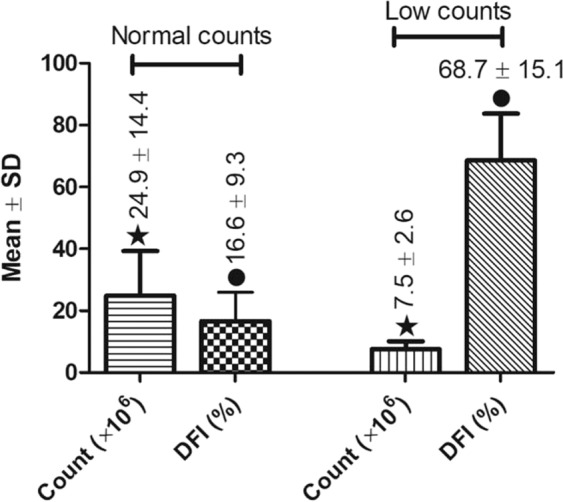
Comparisons of sperm counts and DFI between a group of males with abnormally low sperm counts (n = 10) and a group of males with normal sperm counts (n = 24). P < 0.0001 between two bars with the same symbol.

To determine the correlation between post-thaw sperm DFI and sperm counts, we analyzed sperm from males from 34 mutant strains including 10 with abnormally low sperm counts and 24 with normal sperm counts. Males with lower sperm counts (7.5 ± 2.6 million/male) were associated with a significantly (P < 0.0001) higher DFI (68.7 ± 15.1% vs 16.6 ± 9.3%) than males with normal sperm counts (24.9 ± 14.4%) (Fig. 8).

### Correlation between sperm DFI and fertility

To determine the correlation between DFI and embryo development, we analyzed sperm from mice from 5 mutant mouse strains that failed to produce pups after IVF and embryo transfer (ET) and mice from 5 mutant mouse strains that failed to produce pups after ICSI/ET, and compared to fertile males in both IVF and ICSI groups (Table 1).

**Table 1:** Relationship between frozen-thawed sperm DFI and pup birth rates of embryos derived by IVF or ICSI using frozen-thawed sperm.

	Sperm Count (×10^6^)	Total Motility (%)	Prog. Motility (%)	Normal Heads (%)	Normal Tails (%)	Sperm DFI (%)	2-cell Rate (%)	Embryos transferred	Pup Rate (%)
IVF no pups (n = 5)	20.3 ± 10.2	45.2 ± 9.3	19.0 ± 6.2	70.6 ± 17.4	68.8 ± 11.2	62.7 ± 7.2^*^	14.4 ± 9.4^#^	206	0^&^
IVF Ctrl (n = 7)	19.8 ± 3.8	54.4 ± 16.4	29.7 ± 12.0	74.7 ± 8.4	78.9 ± 3.8	20.4 ± 7.9^*^	32.7 ± 4.9^#^	380	36.4 ± 12.2^&^
ICSI no pups (n = 5)	7.9 ± 3.0^§^	29.6 ± 24.2	3.6 ± 1.9^@^	31.2 ± 16.6^Δ^	50.2 ± 31.7	73.3 ± 8.1^&^	79.6 ± 11.4	188	0^®^
ICSI Ctrl (n = 6)	22.5 ± 7.2^§^	42.2 ± 12.3	21.2 ± 9.1^@^	73.2 ± 6.9^Δ^	71.0 ± 6.6	28.1 ± 10.7^&^	85.8 ± 5.9	240	23.4 ± 6.0^®^

Note: The data with the same symbols in the same column are significantly different (P < 0.05). The total and progressive motilities were obtained before sperm freezing.

IVF rates using sperm from mutant male mice with normal sperm counts, motility, and morphology were lower (14.4 ± 9.4% 2-cell embryos) compared with those using sperm from fertile mutant males (32.7 + 4.9%), and produced no pups after surgical transfer of 206 two-cell stage embryos.

Using sperm from male mice that were unsuitable for IVF and thus necessitated the use of ICSI we also observed lower fertilization rates (79.6 ± 11.4%) compared to fertile mutant males (85.8 ± 5.9%) and obtained no pups after embryo transfer. Further, sperm count, progressive motility, and sperm head morphology in the mutant male ICSI group were poorer than in control males (P < 0.05). However, sperm DFI of mutant males that failed IVF and ICSI (62.7 ± 7.2% and 73.3 ± 8.1%, respectively) was significantly higher (P < 0.0001) than that of control males used for IVF and ICSI (20.4 ± 7.9% and 28.1 ± 10.7%, respectively). In addition, the fertilization rate (2-cell rate) after IVF was significantly less (P < 0.001) using sperm with high DFI (62.7 ± 7.2%) compared to sperm with low DFI (20.4 ± 7.9%).

## Discussion

Sperm cryopreservation has evolved as an important strategy to preserve fertility and facilitate assisted reproduction in animals and humans. Sperm cryopreservation methods include slow freezing, rapid freezing (liquid nitrogen vapor cooling followed by plunging into liquid nitrogen) and vitrification (directly plunging into liquid nitrogen)^33–35^. Although slow freezing is still used for sperm cryopreservation in humans, rapid freezing and vitrification have been found to be more cost-effective, faster and superior for preserving sperm motility and DNA integrity in humans^33–35^. The most popular and efficient sperm cryopreservation technology in mice is rapid freezing using 18% raffinose and 3% skim milk (termed R18S3) plus monothioglycerol (MTG) or glutamine (Glu)^36–38^. Despite the extensive progress that has been made in the field, decreases in sperm motility and DNA integrity are commonly observed after cryopreservation^30,39^. In this report we found that rapid freezing using R18S3 + MTG also significantly reduced sperm motility and increased DNA fragmentation in both wildtype and mutant mouse strains, observations consistent with previous results in mice^26,29^ and humans^30–32,40^. In this report, we also found that DFI of frozen-thawed sperm from mutant mouse strains was consistently and highly correlated with male mouse fertility compared to traditional measures of sperm quality (count, morphology, and motility). This observation is consistent with the perception that sperm nuclear DNA integrity is essential to embryo development^25–27^. Our findings in mice are consistent with results obtained in humans in which sperm with a high proportion of DNA fragmentation were found to be associated with impaired embryo development and increased miscarriage rates^8,10,11,41–44^.

As hypothesized, our data revealed a strong negative correlation between sperm DFI and sperm count, motility and morphology. Our results indicate that sperm DNA integrity as measured by DFI is highly compromised in sperm with extremely low sperm count, poor motility, and/or exhibiting a high proportion of abnormal head morphology. Such strong correlation has also been observed in sperm from human patients suffering from male infertility^43–45^. However, our results indicate that sperm DFI has a higher predictive value than conventional sperm quality assessment parameters for assessing the likelihood of reproductive success. Further, sperm DFI was more predictive than sperm count, morphology, and motility of the success of IVF or ICSI and subsequent litter rates following embryo transfer.

Because sperm DFI is negatively correlated with sperm count and motility, both of which are necessary for sperm to successfully fertilize an oocytes, one would expect an inverse relationship between sperm DFI and fertilization rates. However, our data reveals this is not consistently the case, as has been observed in humans^41,46–49^. Instead, a strong inverse relationship between sperm DNA fragmentation and fertilization is more evident in poor quality sperm samples^45,50,51^. The lack of a consistent correlation between fertilization rate and sperm DFI suggests that blastocyst formation, pregnancy and fetal development might be more negatively impacted than fertilization by sperm DNA damage^25,28,52^. It is known that maternal regulation predominates in the early stage of embryo development, while expression of paternal genes begins at the 4- to 8-cell stage^53^. Therefore, despite a high IVF rate utilizing sperm with a high DFI score, embryo development to term and birth rates can still be negatively impacted^54^.

The significant role of sperm DNA fragmentation as a component in male factor infertility is supported by our results in mice which avoided the marked heterogeneity of human study characteristics (including female factors and cycle characteristics, e.g., day of embryo transfer, etc.). Our results clearly show the importance of testing DNA quality to ensure delivery of an intact genetic payload to oocytes by natural fertilization, intrauterine insemination (IUI), IVF or ICSI. This is particularly emphasized in the case of human reproduction where ICSI is now a routine procedure^55,56^, since ICSI requires only one sperm for the procedure and sperm from infertile men usually have high DFI^57^. Given its relevance to fertility, we believe that DFI used as an assessment of DNA fragmentation should be included as part of sperm quality analysis in humans as well as in animals in order to diagnose infertility, improve outcomes using ARTs, predict fertility, select animals for ART, guide selection of an ART procedure, and increase reproductive success. Our results also show that DFI is especially indicated when a semen sample has poor conventional parameters or in situations of unexplained infertility or recurrent pregnancy loss.

The mostly commonly used SDF tests include comet assay, sperm chromatin structure assay (SCSA), sperm chromatin dispersion (SCD) test (or halo assay) and terminal deoxynucleotidyl transferase-mediated deoxyuridine triphosphate-nick end labeling (TUNEL) assay^57,58^. Comet assay is single-cell gel electrophoresis in which lysed and decondensed sperm are embedded in an agarose gel and subjected to an electrophoretic gradient to separate DNA fragments based on charge and size within individual sperm. Comet assay is labor intensive and is also criticized for underestimation of DNA fragmentation and damaging the alkaline-labile sites^58,59^. SCSA is a flow cytometric test that measures the susceptibility of sperm DNA to acid-induced DNA denaturation *in situ* using a change in fluorescence by acridine orange to differentiate between sperm with fragmented DNA versus those with intact DNA^60^. The disadvantages of SCSA are that it requires expensive equipment for analysis and a DNA denaturation step with the potential to induce DNA breaks in the sperm. The SCD test has been described as a simple and inexpensive method for SDF analysis. It is based on the principle that sperm nuclei with fragmented DNA produce very small or no halos of DNA dispersion that is seen when sperm are embedded in agarose gel following acid denaturation and removal of nuclear proteins^61^. Like SCSA, the SCD test relies on acid denaturation that can potentially induce sperm DNA damage.

The TUNEL assay is considered to be the simplest, most sensitive, accurate and reliable SDF test, and has been most widely used in humans and animals by either flow cytometry or fluorescence microscopy^57,58^. The advantages of the TUNEL assay is that it can measure “true” single and double-stranded DNA breaks by directly incorporating modified nucleotides into the site of damage and does not rely on an additional, potentially damaging denaturation step^62^. Although the TUNEL assay has been used to measure mouse sperm DNA damage^63–65^, strict standardization of the method for use in assessing fertility in genetically altered mice is lacking. Many factors involved in the processing, fixation, and permeabilization of the specimen may affect TUNEL assay results. Based on previous reports in mice and humans^63,66^ and our data presented here, we believe that a reproducible TUNEL assay uses (1) EDTA as an inhibitor of sperm endonuclease activity to block additional production of sperm DNA fragmentation during sperm processing^63^, and (2) dithiothreitol (DTT) or β-mercaptoethanol to reduce the disulphide linkage between protamine molecules, relaxing the chromatin and thereby allowing TdT to access the DNA strand breaks within the sperm nucleus^63,66^ to avoid underestimating DNA damage. The results presented in this report indicate that our TUNEL assay protocol is a simple, reliable, and reproducible method to assess sperm DNA damage in mice. It is important to point out, however, that intraspecific differences between sperm structure and chromatin chemistry should guide the selection of the most appropriate technique for assessment of DNA fragmentation in animals, including humans^55^. Thorough validation and development of species-specific protocols are essential when developing such assays to ensure safety and reproducibility of analytical results.

In summary, we have demonstrated that present-day laboratory methods of sperm analysis using conventional parameters are a poor predictor of reproductive outcome. DFI is a simple means to more reliably, accurately, and reproducibly assess sperm quality and fertility and for predicting the likelihood of fertilization and littering success when using frozen-thawed sperm for ART. In addition, sperm DFI is useful for determining sperm DNA integrity as a means to accurately assess the fertility of male mice irrespective of their sperm count, motility, and/or morphology. Further, evaluation of sperm DNA fragmentation can be used to select male mice for successful rederivation, sperm cryopreservation and cryorecovery of genetically modified mouse strains. Finally, sperm DFI can help identify causes of unexplained male infertility or failure to litter after surgical transfer of IVF and/or ICSI-derived embryos. The results of the present study indicate that SDF testing should be included in the evaluation of male factor fertility along with standard semen analysis in humans and animals.

## Materials and Methods

### Mice

Three to 5 month old mutant male mice from 83 genetically-modified mouse strains on a C57BL/6N genetic background were obtained from the KOMP Repository (www.komp.org) and the MMRRC at UC Davis (mmrrc.ucdavis.edu). Male and female wildtype C57BL/6N mice were purchased from Charles River (www.criver.com) to serve as donors of control sperm and oocytes, respectively. All mice were fed *ad libitum* a standard mouse chow diet and maintained in temperature and light-controlled rooms (23 °C, 14 hours light: 10 hours dark) prior to use. Euthanasia was performed by CO_2_ asphyxiation followed by cervical dislocation according to the 2013 AVMA guidelines. All animal use was conducted in accordance with the Animal Welfare Act. Studies were performed consistent with the ILAR 8th Revision to the Guide for the Care and Use of Laboratory Animals and in compliance with and with prior approval from the UC Davis institutional animal care and use committee (IACUC protocol # 20863).

To study the effects of cryopreservation on sperm motility and DNA integrity (Result 1, Fig. 1), sperm from 24 wildtype C57BL/6N male mice and mutant males for 24 fertile mutant mouse strains were used.

To study the correlation between sperm DFI and motility in mutant males (Result 2, Figs. 2, 3), sperm from 60 mutant mouse strains were used. In addition, sperm from 24 fertile mutant males were compared with that of 17 mutant males with impaired motility (Fig. 4).

To study the correlations between post-thaw sperm DFI and sperm morphology in mutant males (Result 3, Figs. 5, 6), sperm from 60 mutant mouse strains mentioned above were used. In addition, sperm from the 24 fertile mutant males mentioned previously were compared with sperm from 9 mutant mouse strains (Fig. 7).

To study the correlation between post-thaw sperm DFI and sperm count in mutant males (Result 4, Fig. 8), sperm from 10 mutant mouse strains with abnormally low sperm count and that from 24 fertile mutant mouse strains with proven fertility mentioned above were used.

To study the correlation between sperm DFI and fertility (Result 5, Table 1), sperm from 10 mutant mouse strains were used for IVF (5 males) and ICSI (5 males). Sperm from fertile mutant males with normal fertility were used as the controls for IVF (n = 7) and ICSI (n = 6).

### Sperm cryopreservation

Sperm freezing medium R18S3 + MTG (18% raffinose, 3% skim milk and 477 µM α-monothioglycerol in water) was used for sperm cryopreservation as described previously^36,37^. Briefly, after euthanasia of male mice both cauda epididymides were dissected and cleaned of blood and fat under a dissecting microscope and then placed into 0.5 mL freezing medium in a Falcon center well organ culture dish. A syringe needle (26 G) was used to gently pierce and express sperm from the cauda epididymides into freezing medium. After incubation for 10 min at 37 °C, the sperm suspension was mixed and divided into two parts: One part was analyzed immediately for sperm DNA integrity assessment and conventional parameters (count, motility and morphology), and the other part was cryopreserved in 1.8 mL cryotubes (50 μL each tube, NUNC 377267, 8 samples per male) by rapid freezing in liquid nitrogen vapor (−130 °C) for 10 min followed by full submersion in liquid nitrogen. After at least 1 week of storage in liquid nitrogen, sperm samples were thawed at 37 °C for 5 min in a water bath and analyzed immediately for DFI and conventional parameters.

### Sperm count and motility

An IVOS II computer-assisted sperm analyzer (Hamilton Thorne, Beverly, Massachusetts) was used to assess sperm count and motility of each sample after dilution in M2 medium at 1:19 ratio using a wide-bore pipet tip. Sperm count was calculated by sperm concentration (millions/mL) × 0.5 mL. Sperm total motility (% of sperm displaying any type of movement) and progressive motility (% of progressively swimming sperm with average path velocity ≥50 μm/s and straightness ratio ≥50%) were analyzed in chambers with depth 80 μm (2X-cel slides, Hamilton Thorne) by reading 8–10 fields (2000–3000 sperm) in two counting chambers at 37 °C as described previously^24,37^.

### Sperm head and tail morphology

Sperm morphology was assessed for each male. A smear of each sperm sample was air-dried on 2X-cel slide and assessed morphologically at 400x magnification under a phase contrast microscope. A minimum 200 sperm from >5 fields were counted for percent of sperm with normal head morphology, and then a minimum 200 sperm from >5 fields were counted for percent of sperm with normal tail morphology. Abnormal heads included any abnormal sizes and shapes, such as macrocephalous, microcephalous, tapered, triangular, olive, pin, banana, amorphous, collapsed, abnormal hook, irregularly shaped, etc. Abnormal sperm tails included both midpiece and tail abnormalities, such as bent, coiled, short, thin, crinkled, irregularly shaped, etc.

### Sperm TUNEL assay

**Figure 9:**
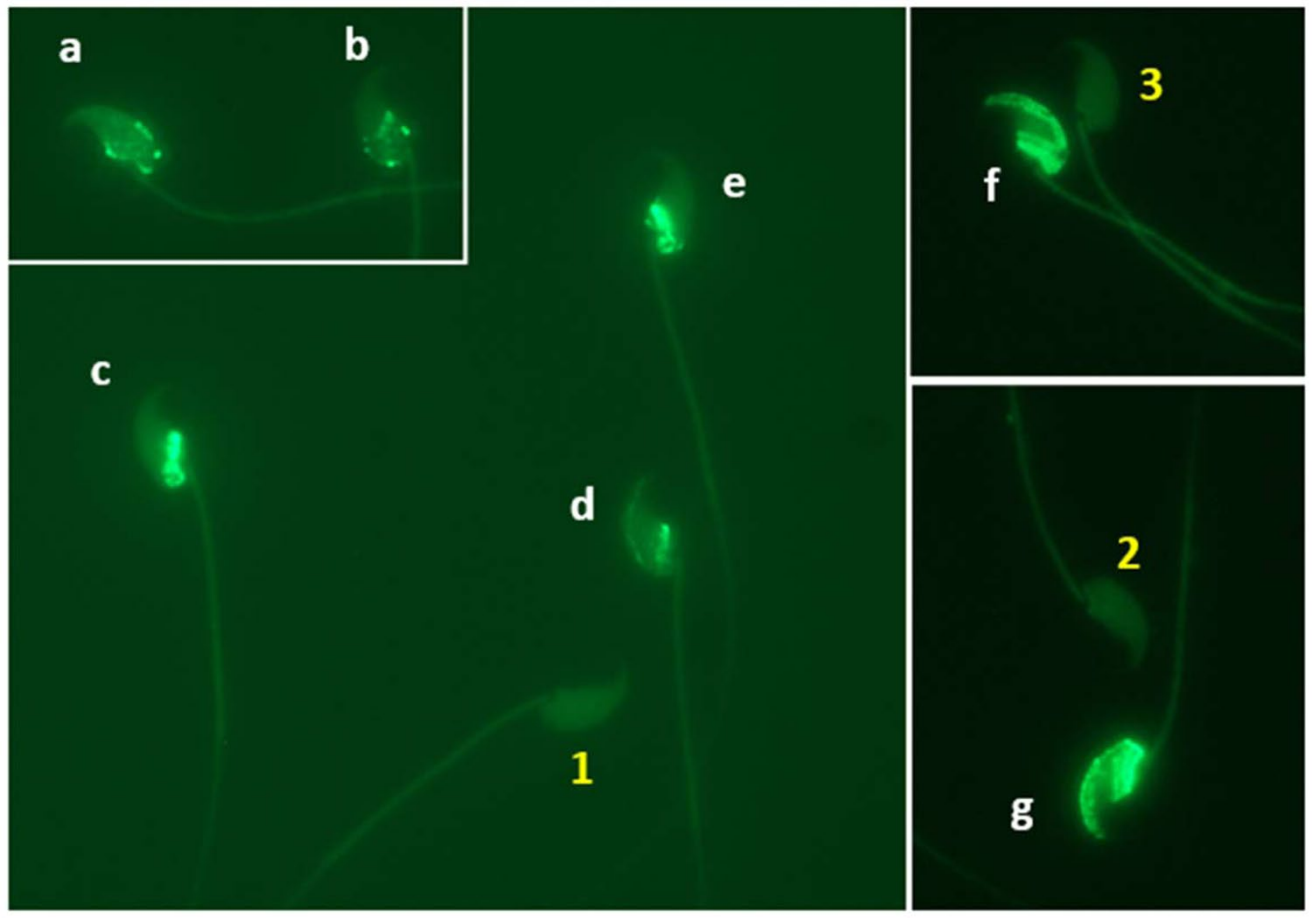
TUNEL negative (1, 2, and 3) and positive (**a–g**) labeled sperm indicating intact and fragmented nuclear DNA, respectively. Original magnification 1000×.

Sperm DFI was assessed for each male. A suspension of sperm sample was mixed with 1% low melting agarose in Ca/Mg-free Dulbecco’s phosphate-buffered saline (DPBS) at 37 °C at a 3:7 ratio by gently pipetting using a wide-bore pipet tip. Fifty µL of each sperm-agarose mixture was pipetted onto a sterile glass slide pre-coated with 0.65% normal melting agarose and spread by covering with a sterile 22×40mm  coverslip. Loaded slides were placed at 4 °C for 5 min to allow the agarose to produce a microgel with sperm cells trapped within it. Afterward, the cover glass was removed by sliding it off gently from each slide which was then immersed in DPBS containing 5 mM dithiothreitol (DTT) and 1 mM EDTA for 30 min on ice to decondense sperm nuclei. After washing with ice-cold DPBS, the sperm were fixed in ice-cold 4% (w/v) paraformaldehyde in DPBS overnight followed by storage in 70% ethanol at −20 °C for 1 week before being washed in DPBS and permeabilized in 0.5% Triton X-100 in DPBS for 10 min at room temperature. Then the sperm on the slide was washed in DPBS, and sperm DNA was nick-end-labeled with FITC conjugated dUTP using the *In Situ* Cell Death Detection Kit (Roche Molecular Biochemicals, Mannheim, Germany). Briefly, 50 µl DNA labeling reaction was added onto each slide, spread by covering a sterile cover glass, and then incubated in a humidified incubator at 37 °C for 1 hour in the dark. Slides were then washed in DPBS after gently removing the cover glass and mounted with VECTASHIELD antifade mounting medium with DAPI (4,6 diamidoino-2-phenylindole) (Vector Laboratories, Burlingame, CA, USA) to stain sperm DNA. A minimum of 200 sperm per sample from at least 5 fields selected in a blinded fashion were analyzed at 1000× magnification under a Nikon Eclipse Ci-FL fluorescence microscope with a standard fluorescein filter set. The total number of sperm per field stained with DAPI (blue) was first counted, and then the number of cells labeled with intense green fluorescence (TUNEL positive showing DNA fragmentation, see Fig. 9) was counted. The DFI was calculated as the percentage of TUNEL positive sperm (=[TUNEL positive/(TUNEL positive + TUNEL negative)] × 100%).

### IVF, ICSI and embryo transfer

For each male, IVF or ICSI using frozen-thawed sperm followed by embryo transfer was performed to correlate the DFI score for each sperm sample with its fertilization potential and likelihood for producing viable offspring. To avoid female factor infertility, wildtype oocytes from C57BL/6NCrl female mice were used for all IVF and ICSI procedures. Four weeks old (for IVF) or 7–10 weeks old (for ICSI) female C57BL/6N mice were superovulated by intraperitoneal (IP) injection of 5 IU of pregnant mare serum gonadotropin (PMSG) followed 47 h later by IP injection of 5 IU of human chorionic gonadotropin (hCG). Each IVF or ICSI procedure used sperm from 1 male and oocytes from at least 5 females. Cumulus-oocyte-complexes (COCs) were collected following euthanasia 14–15 hours post hCG injection. For ICSI, oocytes were dissociated from cumulus cells by treatment with 300 U/ml bovine testis hyaluronidase in M2 medium at 37 °C, and then washed and cultured in pre-equilibrated KSOMaa medium at 37 °C in humidified 5% CO_2_ in air.

IVF was performed in 5.5% CO_2_ in humidified air at 37 °C using a modification of a previously published MBCD-GSH IVF method^24,37^. Briefly, thawed sperm were mixed and distributed evenly into 3 pre-equilibrated 120-µL long-flat MBCD medium drops (10 µL sperm per drop), and then incubated for 50–60 min before IVF. COCs collected from 15 superovulated females were pooled, mixed and distributed evenly into 300 µL drops of RVF medium containing 0.8 mM reduced glutathione (GSH) pre-equilibrated in CO_2_ incubator. After sperm had been incubated in the MBCD medium for 50–60 min, total 45 µL sperm collected from the 3 MBCD medium drops (15 µl from each drop) was added to each IVF drop. After 4 hours of co-culture, oocytes were washed and incubated in RVF medium overnight until the next morning when dishes were scored for IVF rate (number of 2-cell embryos/total number of oocytes used).

ICSI was performed in M2 medium at room temperature using modifications of a method described previously^67^. Briefly, sperm heads were separated from tails in clinical grade polyvinylpyrrilodone (PVP) solution (LifeGlobal Group: www.lifeglobalgroup.com) by piezo vibrations and then injected into oocytes. Injected oocytes were washed and incubated in equilibrated KSOMaa medium at 37 °C in humidified 5% CO_2_ in air overnight until 2-cell stage.

Two-cell stage embryos derived by either IVF or ICSI were surgically transferred into the oviducts (10–13 for each oviduct, 20–25 per recipient) of pseudopregnant CD-1 mouse recipients 0.5 day post coitum anesthetized with an intraperitoneal injection of 0.01 mL/g body weight of ketamine/xylazine solution (10 mg/mL ketamine and 1 mg/mL xylazine). Immediately after surgery all mice received a subcutaneous injection of 0.1 mL of pain reliever Buprenex (0.03 mg/mL; Western Medical Supply, Inc., Arcadia, CA, USA). Recipients were kept warm on a heating pad until fully recovered from anesthesia. All pregnant recipients were allowed to go to term and give birth to litters.

### Statistical analyses

GraphPad Prism 8 software (GraphPad Software, Inc., San Diego, California) was used for statistical analyses. Sperm motility, percentages of sperm with morphologically normal heads or tails, sperm DFI, and embryo transfer pup birth rates were arcsine transformed, and then significant differences between treatments were detected by one-way ANOVA followed by Tukey HSD tests or t tests. Data are expressed as mean (M) ± standard deviation (SD). The strengths of the linear correlation between variables were also measured using the software. The level of significance was set at P < 0.05.
